# Streptomycin Sulfate–Loaded Niosomes Enables Increased Antimicrobial and Anti-Biofilm Activities

**DOI:** 10.3389/fbioe.2021.745099

**Published:** 2021-10-27

**Authors:** Maryam Mansouri, Nazanin Khayam, Elham Jamshidifar, Tara Pourseif, Sepideh Kianian, Amir Mirzaie, Iman Akbarzadeh, Qun Ren

**Affiliations:** ^1^ Department of Microbiology, Faculty of Advanced Science and Technology, Tehran Medical Sciences, Islamic Azad University, Tehran, Iran; ^2^ Department of Biology, Tehran North Branch, Islamic Azad University, Tehran, Iran; ^3^ Department of Pharmaceutical Nanotechnology, Faculty of Pharmacy, Tehran University of Medical Sciences, Tehran, Iran; ^4^ Master of Medicinal Chemistry, Medicinal Plants and Drugs Research Institute, Shahid Beheshti University, Tehran, Iran; ^5^ Department of Biology, Parand Branch, Islamic Azad University, Parand, Iran; ^6^ Department of Chemical and Petrochemical Engineering, Sharif University of Technology, Tehran, Iran; ^7^ Laboratory for Biointerfaces, Empa, Swiss Federal Laboratories for Materials Science and Technology, St. Gallen, Switzerland

**Keywords:** niosome, streptomycin sulfate, antimicrobial, anti-biofilm, cytotoxicity

## Abstract

One of the antibiotics used to treat infections is streptomycin sulfate that inhibits both Gram-negative and -positive bacteria. Nanoparticles are suitable carriers for the direct delivery and release of drug agents to infected locations. Niosomes are one of the new drug delivery systems that have received much attention today due to their excellent biofilm penetration property and controlled release. In this study, niosomes containing streptomycin sulfate were prepared by using the thin layer hydration method and optimized based on the size, polydispersity index (PDI), and encapsulation efficiency (EE%) characteristics. It was found that the Span 60-to-Tween 60 ratio of 1.5 and the surfactant-to-cholesterol ratio of 1.02 led to an optimum formulation with a minimum of size, low PDI, and maximum of EE of 97.8 nm, 0.27, and 86.7%, respectively. The drug release investigation showed that 50.0 ± 1.2% of streptomycin sulfate was released from the niosome in 24 h and reached 66.4 ± 1.3% by the end of 72 h. Two-month stability studies at 25° and 4°C showed more acceptable stability of samples kept at 4°C. Consequently, antimicrobial and anti-biofilm activities of streptomycin sulfate–loaded niosomes against *Staphylococcus aureus*, *Escherichia coli*, and *Pseudomonas aeruginosa* were found significantly higher than those of free drug, and the minimum inhibitory concentration values decreased 4- to 8-fold. Furthermore, niosome-encapsulated streptomycin up to 1,500 μg/ml exhibited negligible cytotoxicity against the human foreskin fibroblasts cell line, whereas the free drug exhibited slight cytotoxicity at this concentration. Desired physical characteristics and low toxicity of niosomal nano-carriers containing streptomycin sulfate made them a demanded candidate for the treatment of current bacterial infections and biofilms.

## Introduction

The use of drug delivery systems is essential to improve the timing, location, and speed of drug release, as well as to prevent drug fluctuations in the circulatory system that lead to lower efficacy and greater side effects (Vikas et al., 2011; Shirzad et al., 2019; Shad et al., 2020). Progress in nanotechnology led to the development of nano-carriers that are able to carry drugs to the target site (Ladavière and Gref, 2015; Heidari et al., 2020). Nano-carriers have benefits such as increased drug solubility, increased drug half-life, controlled release, targeted delivery, reduced side effects, the ability to transfer multiple drugs simultaneously, protecting the drug from degradation, and protecting the patient from immune responses to the drug (Zhang et al., 2008; Banyal et al., 2013; Ghafelehbashi et al., 2019; Akbarzadeh et al., 2021a).

One of the types of nano-carriers is niosomes, which can be an ideal choice because they are biocompatible, inert, and capable of carrying large dosage of one or more drugs (Lajevardi et al., 2018; Fang et al., 2019). They are composed of non-ionic surfactants of the class alkyl or polyglycerol diallyl ether and cholesterol, hydrated in aqueous media (Kumar and Rajeshwarrao, 2011), and can be used as carriers for low–molecular weight drugs, proteins, and genes. Due to the numerous benefits of niosomes as a drug carrier, much research has been carried out and proven to be effective for drug delivery to skin, ocular, oral, and pulmonary sites (Kumar and Rajeshwarrao, 2011; Akbarzadeh et al., 2020a; Akbarzadeh et al., 2020c). Some diseases, such as bacterial infections, require a high dosage of medication with controlled release. Also depending on the condition of these patients, it is important to have the least side effects and decrease drug resistance (Zhang et al., 2008; Banyal et al., 2013; Reta et al., 2019). Therefore, niosomal drug carriers can be a suitable candidate for infectious diseases (Akbari et al., 2013; Akbarzadeh et al., 2020c).

Infectious diseases are a major health problem and are one of the leading causes of death in developing countries. In infection, the body is invaded by pathogenic microorganisms which establish, grow, and proliferate in the host body, leading to localized cell damage, toxin secretion, or antigenic antibody responses (Brunner, 1970; Glanze et al., 1990; Bloom et al., 2000; Reta et al., 2019). Most of the commonly used antibiotics are becoming inefficient against pathogenic bacteria because of biofilm formation. Biofilms are complex structures of aggregate bacteria which are capable to survive in stressful environment conditions and cause antibiotic resistance. In recent years, different types of nanoparticles have been developed for biofilm treatment (Samiei et al., 2016; Shrestha and Kishen, 2016; Fulaz et al., 2019; Mirzaie et al., 2020).

Streptomycin is a broad-spectrum antibiotic because it kills both Gram-negative and Gram-positive bacteria and was one of the first aminoglycoside drugs discovered. It cannot be taken orally and is often prescribed as regular intramuscular injections. Aminoglycosides block the protein synthesis in the bacterium by binding to the S12 protein of the 30 S ribosomal unit (Judy et al., 2018); thus, they can have a bactericidal effect (Zhu et al., 2001; Sharma et al., 2007; de Lima Procópio et al., 2012).

The purpose of this investigation is developing the streptomycin sulfate–containing niosomes according to the design of experiment, followed by physicochemical characterization. The drug loading and release profiles of the streptomycin sulfate–containing niosomes were investigated. The stability of the prepared niosomes was evaluated at temperatures of 4°C and 25°C for 3 months in terms of nanoparticle size, particle size distribution (PDI), and drug encapsulation efficiency (EE%). Finally, the antimicrobial and anti-biofilm effects of niosomes containing streptomycin sulfate on microbial strains of *Staphylococcus aureus*, *Escherichia coli*, and *Pseudomonas aeruginosa* were compared with the free drug.

## Materials and Methods

### Chemicals

Streptomycin sulfate and phosphate buffer solution (PBS) were purchased from Bio Basic, Canada. Cholesterol, polyoxyethylene sorbitan monostearate (Tween 60), sorbitan monostearate (Span 60), dimethyl sulfoxide (DMSO), and chloroform were bought from Merck, Germany. 3-(4,5-dimethylthiazol-2-yl)-2,5-diphenyltetrazolium bromide, penicillin/streptomycin 100X, Trypsin-EDTA, Trypan blue, RPMI 1640 medium, Dulbecco’s modified Eagle medium (DMEM), fetal bovine serum (FBS) were obtained from Gibco, United States. A dialysis membrane (MWCO 12,000 Da) and MTT (dimethylthiazol-2-yl-)-2,5 were received from Sigma-Aldrich (United States). Mueller Hinton broth, Mueller Hinton agar, barium chloride, and H_2_SO_4_ were received from Merck, Germany. *Staphylococcus aureus* ATCC 6538, *Escherichia coli* ATCC 25922, and *Pseudomonas aeruginosa* ATCC 15442 were obtained from the Pasteur Institute of Iran.

### Preparation of Niosome

One of the most known methods for preparing niosomes is the thin-layer hydration method (Hülsermann et al., 2009; Akbarzadeh et al., 2021b). Cholesterol and surfactants (Span 60 and Tween 60) with a 1:1 M ratio were dissolved in 10 ml chloroform, which was evaporated using the rotary evaporator for 1 h at 60°C and 120 rpm. Afterward, the dried thin films were hydrated using streptomycin sulfate solution in PBS (10 ml, 1.5 mg/ml) at 30°C for 1 h with stirring at 120 rpm. Subsequently, the samples were sonicated (Hielscher UP50H ultrasonic processor, Germany) for 5 min and stored at 4°C in a refrigerator. Different formulations of niosomes were prepared, as shown in Table 1.

**TABLE 1:** Composition of different formulations of niosomes.

Drug	Formulation	A: Span 60:Tween 60	B: Surfactant:cholesterol	Z average	PDI	EE
	Unit	Molar ratio	Molar ratio	nm	—	%
Streptomycin sulfate (S)	S_1_	0.5	1	85.9	0.40	77.7
	S_2_	0.5	2	146.2	0.24	77.3
	S_3_	0.5	3	203.2	0.11	64.4
	S_4_	1.0	1	65.5	0.30	82.4
	S_5_	1.0	2	98.2	0.25	79.4
	S_6_	1.0	2	129.1	0.25	80.1
	S_7_	1.0	2	92.5	0.25	79.8
	S_8_	1.0	3	170.4	0.31	68.2
	S_9_	1.5	1	125.0	0.27	85.7
	S_10_	1.5	2	159.3	0.25	82.1
	S_11_	1.5	3	362.0	0.29	71.2

### Optimization of Synthesized Niosome by Design of Experiments

The purpose of applying the design of experiments is to identify the factors influencing the experiment process and determine the optimal values. The design method used in this study is D-optimal design using Design-Expert 7.0.10 software (Stat-Ease Inc., United States). This technique can identify the variables that have the most impact on output and evaluate the most optimal conditions in terms of effective factors (Gunst, 1996; Bernkop-Schnürch et al., 2006). For this purpose, two factors were considered: the surfactant-to-cholesterol ratio and the Span 60-to-Tween 60 M ratio as test variables, and the nanoparticle size, polydispersity index (PDI), and encapsulation efficiency (% EE) as test responses. These variables were selected on the basis of information obtained from previous studies (Moghtaderi et al., 2021) and initial screening tests.

The morphology of optimized niosomes was characterized by the field emission scanning electron microscope (SEM). For imaging, the nanoparticle suspension was diluted 1:100 in deionized water; a drop of sample was spread on a conductor film such as aluminum and dried at room temperature.

### Physicochemical Characterization

#### Particle Size

A dynamic light dispersion analysis is a fast, non-destructive physical method used to determine the size of particles in solution and depends on the interaction of light with the particle. Therefore, Zetasizer (Malvern Instrument Ltd. Malvern, the United Kingdom), equipped with a green laser with a wavelength of 633 nm, was used to evaluate the particle size at 25°C. The particle size is the mean particle diameter which is represented as Z-average in nanometers. Accordingly, the more the Z-average, the larger will be the particle size.

#### Polydispersity Index

The degree of particle scattering indicates the degree of dissimilarity of the particle size distribution, calculated by the Malvern nanosizer (Malvern Instrument Ltd. Malvern, United Kingdom) based on the following formula:

PDI=Mw/Mn.

#### Encapsulation Efficiency

To separate the free drug from the niosome-encapsulated drug, the niosomes were centrifuged at 4°C at 14,000 g for 30 min. Through this process, the niosomes are precipitated and free drug remains in supernatant. The amount of streptomycin sulfate in the supernatant can be quantified by measuring the absorbance at 560 nm wavelength (Blainski et al., 2013), using a calibration curve. Finally, by applying the following formula, the percentage of encapsulation efficiency was calculated:

$$\text{Encapsulation efficiency}\%\;=\frac{\text{the amount of}\;\text{initial Streptomycin }-\text{Streptomycin in supernatant}}{\text{the amount of initial Streptomycin }}\times 100.$$

#### *In Vitro* Drug Release Kinetics

In order to evaluate the amount of drug released from niosomal carriers over a specified period of time, the dialysis membrane (molecular weight cutoff 12 KDa) was used to separate noisomes from the free drug. The dialysis bags containing niosomes (2 ml of the samples prepared earlier) or streptomycin (1.3 mg/ml) were placed in 50 ml of PBS solution (pH 7.4), which was under constant magnetic stirring at 37 ± 1°C. At desired time points (1, 2, 4, 8, 24, 48, and 72 h), 1 ml solution was sampled and replaced with 1 ml of fresh PBS solution. The collected sample solution was measured by colorimetric assay for the absorbance at 560 nm *via* UV-vis spectrophotometry (Jasco V-530, Japan Servo Co. Ltd., Japan) (Aman et al., 1995). To study the release kinetics and the mechanism of drug release from the niosomal formulation, the data of the drug release were mathematically analyzed based on the proportional models in kinetic models’ equations, including zero-order kinetics, the Higuchi model, first-order kinetics, and the Korsmeyer–Peppas equation, by using linear form diagrams.

### Storage Stability Studies

To investigate storage stability of the synthesized niosomes, 1 ml of the streptomycin sulfate–loaded niosome solution with 1 mg/ml drug concentration was poured into glass vials and stored at 4 and 25 °C, respectively, for 1 month. Samples were evaluated for the particle size and EE% at different time intervals.

#### Antimicrobial Activity

The minimum inhibitory concentration (MIC) and minimum bactericidal concentration (MBC) were performed for empty niosome, drug-loaded niosome, and free drug using a classical microdilution method. The samples were diluted with Mueller Hinton broth (MHB), of which 100 µl was added to a well of 96-well microplate. Furthermore, the inoculums were prepared at 0.5 McFarland’s standard and 50 µl of selected bacterial culture including *S. aureus* (ATCC 6538), *E. coli* (ATCC 25922), and *P. aeruginosa* (ATCC 15442) was added to each well. Finally, the samples were incubated overnight at 37 °C, and the absorbance of each well was read using a microplate reader at 600 nm (Fard et al., 2018; Ghomi et al., 2020). The lowest concentration at which no growth was observed was considered as MIC.

To assess the MBC values, 10 µl from each well was spread on Mueller Hinton agar and incubated overnight at 37°C. Afterward, colonies were counted, and MBCs were defined as the lowest concentration of samples required to kill bacteria.

#### Time-Kill Assay

Antibacterial activity of free and streptomycin sulfate–loaded niosomes was determined against *S. aureus*, *E. coli*, and *P. aeruginosa* within 72 h using a microplate technique (Sadeghi et al., 2019). In brief, 100 µl of the samples (free streptomycin and noisome-encapsulated streptomycin) in their sublethal concentrations (half of the MIC) were added into the 96-well microtiter plate which was preloaded with 100 µl of each bacterial suspension having 10^5^ CFU/ml. After incubation at 37°C, optical density at OD 600 nm was measured at 2, 4, 6, 24, 48, and 72 h using a microplate reader (EPOCH, Japan).

#### Anti-Biofilm Activity

The anti-biofilm activity of free and niosome-encapsulated streptomycin sulfate against biofilms of *S. aureus*, *E. coli*, and *P. aeruginosa* was done using a microtiter plate–based crystal violet (CV) assay (Behdad et al., 2020). First, 180 μl of Mueller Hinton broth (MHB) culture medium and 20 μl of pathogenic bacteria were added to each well to an OD 600 at 0.6, and the mixture was incubated for 48 h at 37°C at 120 rpm to allow biofilm formation. Then 100 μl of niosomal streptomycin and free streptomycin at the MIC level, free niosome, and free MHB medium (negative control) were added. The plates were then incubated at 37°C for 24 h. Afterward, the supernatants were removed and the wells washed with 300 μl PBS to remove non-adherent cells from the wells. The plates were then air-dried. The biofilms were fixed with 175 μl 2% sodium acetate and stained with 175 μl 0.1% violet crystal for 30 min in the dark. The wells were then washed with PBS to remove excess dye. Finally, 200 μl of ethanol was added to the wells, and their absorption was read at 570 nm.

### Cytotoxicity Study

To investigate cytotoxicity of free streptomycin sulfate, free niosome, and streptomycin sulfate–loaded niosome towards the human foreskin fibroblast (HFF) normal cell line, the colorimetric MTT [(3-(4, 5-dimethylthiazol-2-yl)-2, 5-diphenyl-tetrazolium bromide] assay was used. In brief, the HFF cells were seeded into 96-well plates for 24 h at 37°C. Then, various concentrations of free streptomycin sulfate, free niosome, and streptomycin sulfate–loaded niosome were added into each well. After incubation time, 100 µl of MTT dye was added to the wells and incubated for 4 h at 37°C. Subsequently, 100 µl of DMSO was added, and the absorbance was measured at 570 nm using a microplate reader (AccuReader, Metertech, Taiwan), and the cell survival rate was calculated by the formula:

Cell viability(%)= Optical density of sample/Optical density of control ×100.

For control, HFF cells were incubated with the Dulbecco’s modified Eagle medium (DMEM) without the test sample (Ali et al., 2010).

### Statistical Analysis

A Statistical analysis was performed using Design-Expert 7.0.10 software (Stat-Ease Inc., United States) and IBM SPSS Statistics version 24. ANOVA was used to compare multiple samples, and the *p* value < 0.05 was considered significant.

## Results and Discussion

### Niosomal Formulations and Physicochemical Properties

**FIGURE 1:**
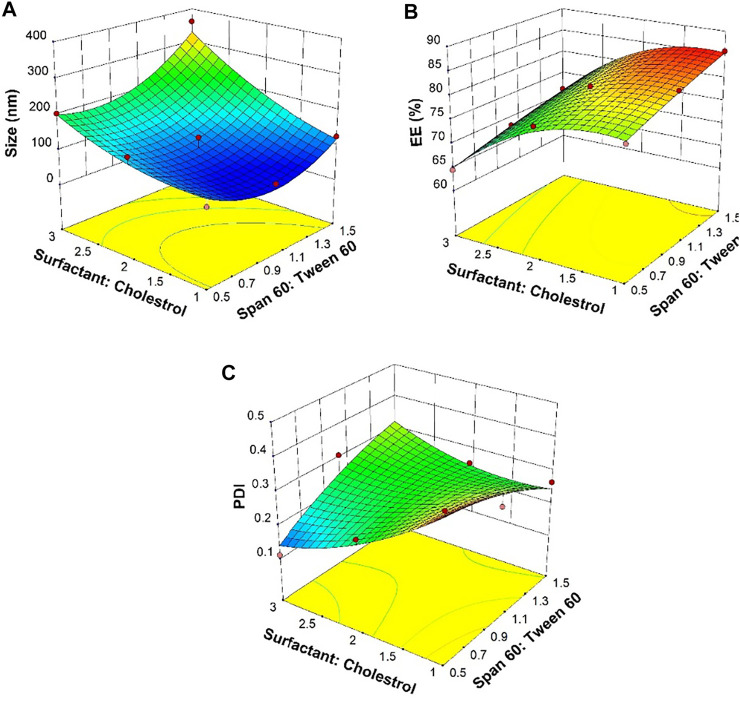
Response surfaces for **(A)** average diameter, **(B)** entrapment efficiency (EE), and **(C)** polydispersity index (PDI) as an outcome of Span 60-to-Tween 60 and surfactant-to-cholesterol molar ratios.

In the structure of the niosomes, the size of the vesicles and the efficiency of streptomycin sulfate encapsulation are highly dependent on the type of surfactant and the ratio of surfactant to cholesterol. For a suitable drug delivery system, it is desired to have a small size and a high encapsulation efficiency (EE) (Moghaddam et al., 2021; Moghtaderi et al., 2021). A variety of niosomal formulations synthesized with different ratios of surfactant to cholesterol and Span 60 to Tween 60 were prepared and compared (Table 1). It was found that with the same Span 60-to-Tween 60 ratio, an increasing surfactant-to-cholesterol ratio led to a larger nano-vesicle size and lower EE. The minimum size was at low levels of the Span 60-to-Tween 60 and surfactant-to-cholesterol ratios as shown in the three-dimensional graph (Figure 1A). The maximum EE of nano-vesicles was found at the high Span 60-to-Tween 60 ratio and low levels of the surfactant-to-cholesterol ratio (Figure 1B). The lowest PDI was found at low Span 60-to-Tween 60 and high surfactant-to-cholesterol ratios (Figure 1C).

As mentioned, Span and Tween are non-ionic surfactants that have many advantages such as improved stability, broad compatibility, and flexibility of formulation. Due to the weak rigidity of Tween 60 and the high lipophilicity of Span 60, proper confinement of cholesterol and surfactant (Span 60:Tween 60) in a 1:1 M ratio can lead to the density of niosome films (Junyaprasert et al., 2012; Ghafelehbashi et al., 2019). By the combination of Span and Tween in different ratios, systems with a wide range of hydrophobic–lipophilic balance (HLB) are produced. In addition, studies have shown that high transition temperatures of Span and Tween 60 provide high levels of drug encapsulation (Bharti et al., 2012; Taymouri and Varshosaz, 2016). Increasing the amount of cholesterol increases the lipid profile and stability of the two layers and results in reduced permeability, so that the drug can be trapped more effectively in the vesicles. However, excessive amounts of cholesterol make the drug and cholesterol compete for the space between the two layers, and consequently, the drug cannot enter the structure (Balakrishnan et al., 2009). It was reported that a 1:1 M ratio of cholesterol to surfactant results in a high EE formulation (Rochdy Haj-Ahmad et al., 2015). The PDI value is an estimate of the particle distribution and their heterogeneity, which is measured between 0 and 1 (Moghassemi et al., 2015). The uniform particles have better distribution and less tendency to accumulate (Waddad et al., 2013).

Based on the data shown in Table 2, multi-criteria optimization was performed by using the desirability function to obtain the optimal formulation (Derringer and Suich, 1980; Esfahani et al., 2019; Shabani et al., 2020). According to the desired parameters, the predicted optimal formulation was calculated and compared with the experimentally obtained one (Table 3). It was found that both predicted and empirically obtained formulation showed similar values, with the latter having a size of 97.8 nm, a polydisperse index of 0.27, and an encapsulation efficiency of 86.7%.

**TABLE 2:** Desirability criteria and predicted values for independent variables.

Number	Span 60:Tween 60	Surfactant:cholesterol	Desirability
1	1.50	1.02	0.80

**TABLE 3:** Comparison of the empirical and predicted values for the optimized niosomal formulation.

Source	Z-average	PDI	EE
Predicted	118.8	0.25	85.5
Empirical	97.8 ± 5.0	0.27 ± 0.03	86.7 ± 1.1

### Morphological Characterization

**FIGURE 2:**
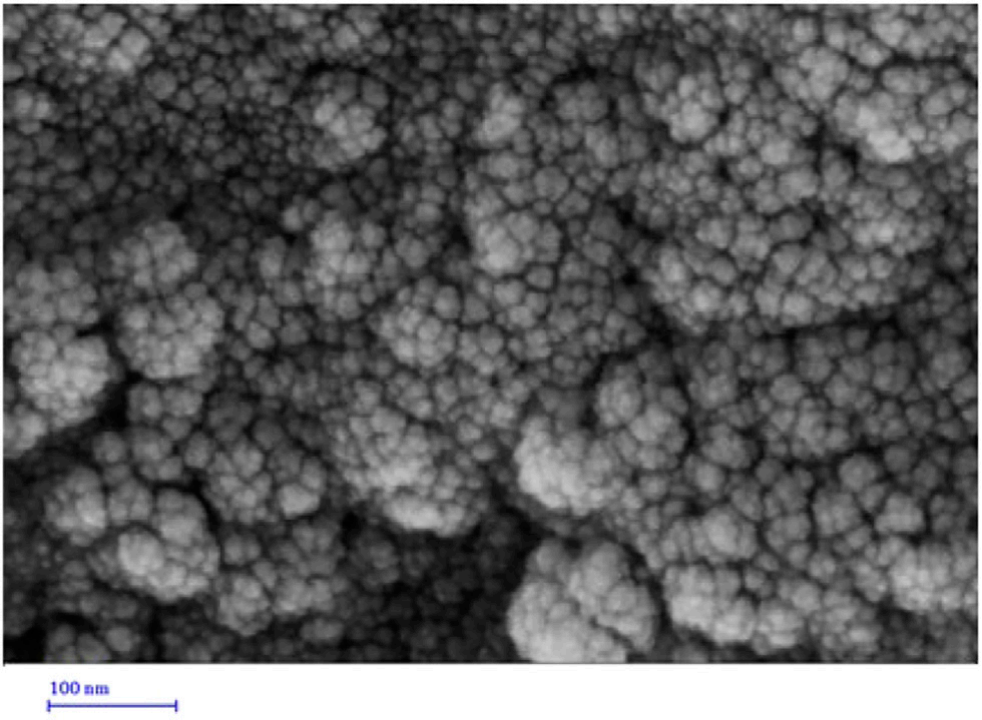
Morphological characterization of optimized niosomes by SEM.

A field emission scanning electron microscope (FE-SEM) was used to investigate the morphology of the synthesized niosomes. It was observed that the streptomycin sulfate–containing niosomes were perfectly spherical in morphology, with a smooth surface (Figure 2). The average particle size for the synthesized niosomes is approximately 20–40 nm, which is less than the size obtained by the light scattering method. This difference could be because the FE-SEM shows the nanoparticle size in the dried form (actual nanoparticle size), while DLS measures the hydrodynamic diameter, which may include any molecule (such as like ions or water molecules) attached to the nanoparticle surface.

Multilayer niosomes were observed using an FE-SEM, which has been reported previously. Niosomes prepared by thin-layer hydration is usually multilayer vesicles below 100 nm with suitable particle distribution that can confine a large amount of drug and slower drug release (Hope et al., 1986; Akbari et al., 2015; El-Sayed et al., 2017).

### Stability Study

**FIGURE 3:**
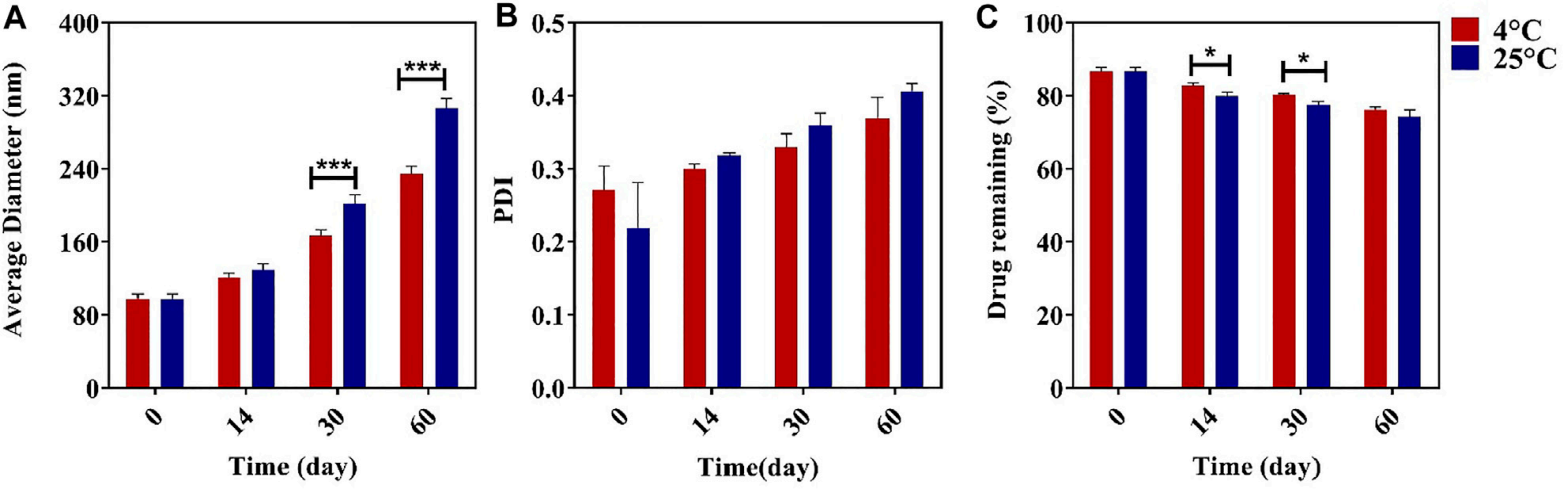
Effect of different temperatures of storage on the average diameter **(A)**, polydispersity index (PDI) **(B)**, and streptomycin sulfate encapsulation efficiency (EE%) **(C)** *n* = 3, *p value < 0.05, **p value < 0.01, and ***p value <0.001.

During storage, the niosomes can swell/break down or are affected by steric/repulsion forces. Here, we investigated the stability of the synthesized niosomes at 4 and 25°C for 60 days. It was found that samples stored at 4°C had better stability in terms of size, PDI, and EE than those stored at 25°C during the 60-day storage (Figure 3). There was a significant difference in the size of the niosomes kept at two temperatures, and the size increase for the samples stored at 4°C was slower than the corresponding sample at 25°C, which could be due to less mobility of the niosomes at 4°C (Lawrence et al., 1996; Balasubramaniam et al., 2002; Akbarzadeh et al., 2020b). Studies have also shown that the size of formulations can affect the stability of the system because, according to the theory of thermodynamics, smaller niosomes contain excess energy, which makes them unstable. The high leakage of the drug at 25°C can also be caused by the higher fluidity of the lipid vesicles at high temperature (Pardakhty et al., 2011; Akbarzadeh et al., 2020b; Akbarzadeh et al., 2020c).

### *In Vitro* Drug Release

**FIGURE 4:**
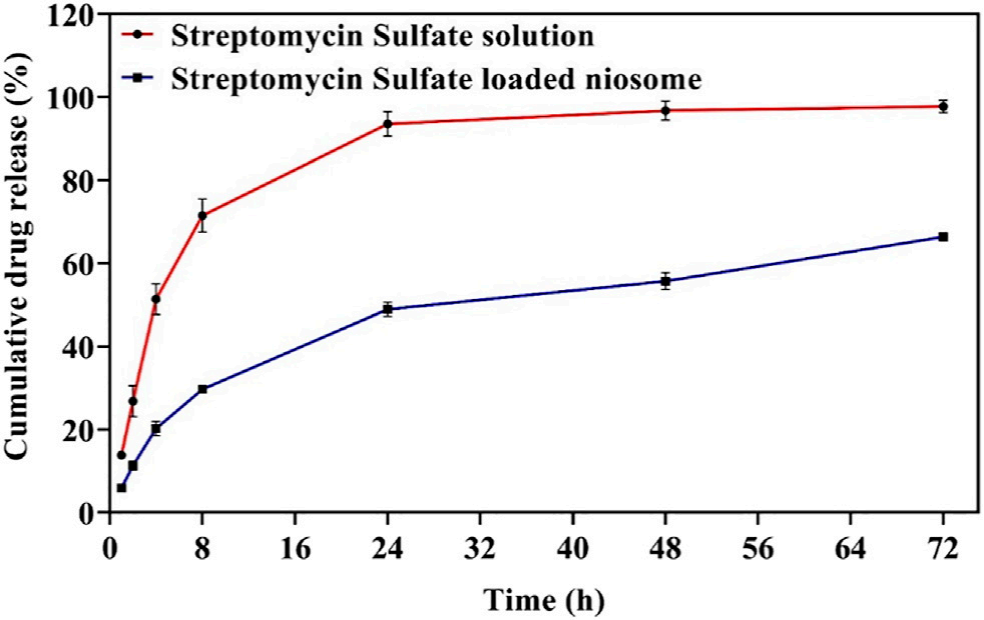
The release profile of free and optimized niosomal formulation of streptomycin sulfate. Each point represents the mean ± SD (*n* = 3).

The drug release rate is an essential factor for upgrading drug delivery systems. The release of encapsulated drugs within the niosomes can be optimized for controlled drug release over the long term (Tarekegn et al., 2010). Here, we compared the release profile of the streptomycin sulfate–soluble form and the encapsulated niosome form in the PBS medium for 72 h. It was found that the streptomycin sulfate release from the nano-carrier (66.4 ± 1.3%) was lower than the drug solution (97.8 ± 1.12%) during 72 h of release (Figure 4). Thus, encapsulation of streptomycin sulfate in the niosome reduced the release burst and allowed more sustainable and prolonged release. It has been previously reported that the release profile of niosomes can have two steps: the first is faster and the second is slower (Paolino et al., 2008; Akbarzadeh et al., 2020c). The rapid release of the drug in the first stage is due to the excretion of the drug from the outer surface of the niosome, and the slower release in the second stage is due to the penetration of the drug through the niosome (Manosroi and Bauer, 1989). Other factors contributing to the release can be components of the niosomes. As the amount of cholesterol in the niosomes increases, the amount of drug release from the vesicles decreases because, at higher cholesterol levels, the movement of the bilayer cannot eliminate osmotic changes and lead to the released drugs absorbed to the niosomal surface (Liang et al., 2004; Ruckmani and Sankar, 2010; Hedayati Ch et al., 2020; Sadeghi et al., 2020). In addition, as the surfactant chain length increases, drug release can continue for a longer period because the transfer temperature can affect the surfactants and make them completely fluid, thereby providing greater penetration of the drug at 37°C (Ruckmani et al., 2000; El-Ridy et al., 2018).

Different models were evaluated to fit the release kinetics of streptomycin sulfate from the optimal niosomal formulation (Table 4). Based on the model parameters and the coefficient of determination (R2) for each model, the release for niosomal formulation was found to follow the Korsmeyer–Peppas model, where the N obtained values (*n* < 0.45) indicate that the Fickian diffusion mechanism determines the release of streptomycin sulfate molecules from the niosomal formulation (Korsmeyer et al., 1983).

**TABLE 4:** Release kinetic models and the parameters obtained for niosomal formulations.

Release model	Equation	*R* ^2^	
		Streptomycin sulfate solution	Streptomycin sulfate–loaded niosome
Zero-order	C_t_ = C_0_ + K_0_t	*R* ^2^ = 0.62	*R* ^2^ = 0.79
Korsmeyer–Peppas	M_t_/M_∞_ = K_t_.t^n^ ^a^	*R* ^2^ = 0.85	*R* ^2^ = 0.94
		*n* = 0.43	*n* = 0.52
First-order	LogC = LogC0+K_t_/2.303	*R* ^2^ = 0.88	*R* ^2^ = 0.85
Higuchi	Q = K_H_ √t	*R* ^2^ = 0.79	*R* ^2^ = 0.92

a Diffusion or release exponent.

### Antimicrobial Activity

**FIGURE 5:**
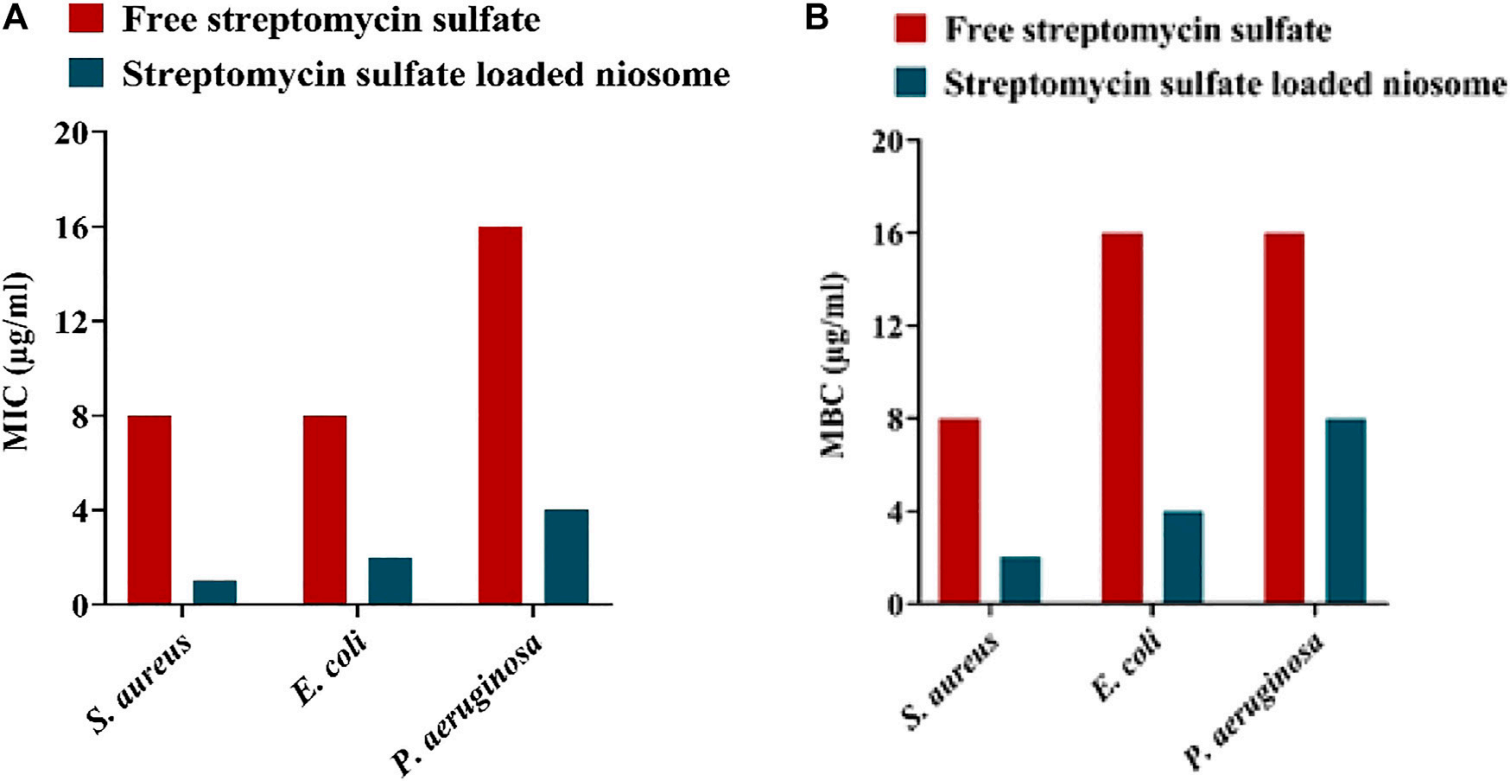
MIC **(A)** and MBC **(B)** of free and niosome-encapsulated streptomycin sulfate. *n* = 3.

We further investigated the antimicrobial activity of the synthesized niosomes by measuring minimum inhibitory concentration (MIC) and minimum bactericidal concentration (MBC). Free niosome, free streptomycin sulfate, and streptomycin sulfate–loaded niosomes against *S. aureus*, *E. coli*, and *P. aeruginosa* were tested. The streptomycin sulfate–loaded niosomes showed a higher antibacterial effect against all studied pathogenic bacteria than free streptomycin sulfate, with the MIC values decreased between 4- and 8-fold (Figure 5). Furthermore, lower MBC was found for the streptomycin sulfate–containing niosomes than for the free streptomycin sulfate. These results suggest that lower concentrations of niosomal streptomycin sulfate are needed to inhibit bacterial growth than free streptomycin sulfate. This could be caused by the possibility that niosomes can protect drug against the effects of bacterial enzymes and facilitate niosome fusion with the bacterial membrane, as reported previously (Mugabe et al., 2005; Moammeri et al., 2021; Moghtaderi et al., 2021).

**FIGURE 6:**
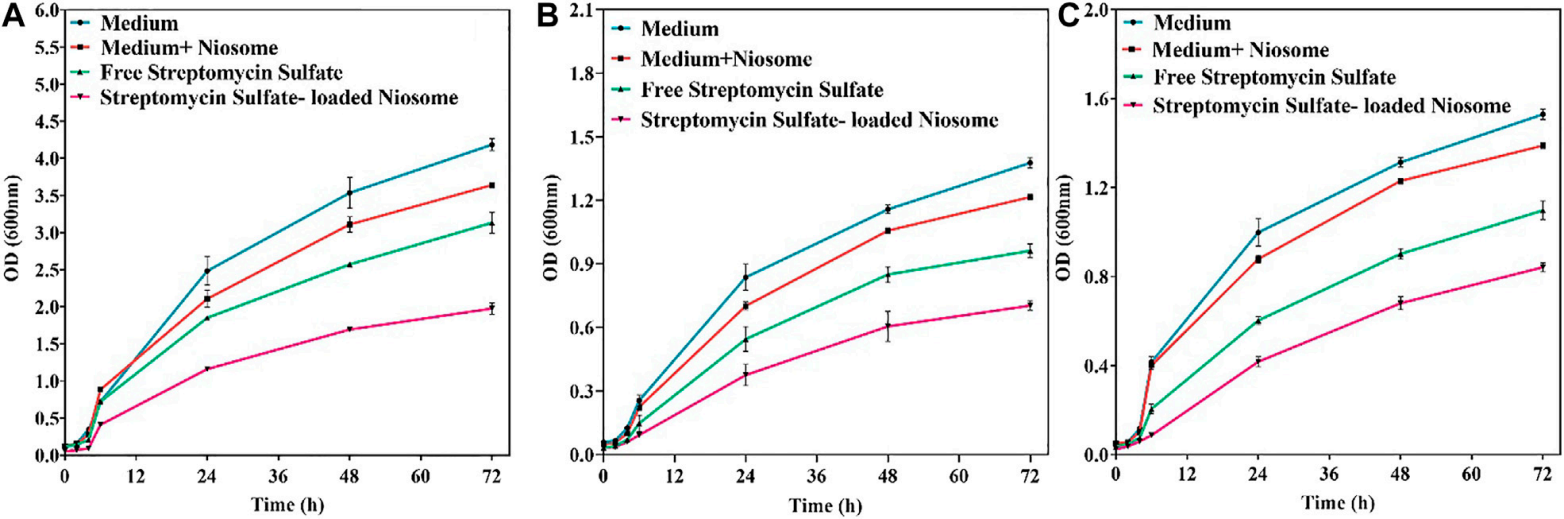
Antibacterial activity of free streptomycin and encapsulated streptomycin against pathogenic bacteria: *S. aureus* **(A)**, *E. coli* **(B)**, and *P. aeruginosa* **(C)** measured by optical density as a function of time (72 h). Each point corresponds to a mean ± SD with three replicates per condition.

To investigate the killing profile of the synthesized niosomes, we next performed the time-kill assay against *S. aureus*, *E. coli*, and *P. aeruginosa* with sublethal concentrations (half of the MIC shown in Figure 5). During the 72-h test, the loaded niosomes displayed highest antibacterial activity compared to the unloaded niosomes and free streptomycin (Figure 6). The results further demonstrate that the direct interaction of the niosomal carrier with bacteria (likely cell membrane) could be a reason for the greater antibacterial property in the niosomes, as reported previously (Zille et al., 2015; Raza et al., 2016; Ghafelehbashi et al., 2019; Moghtaderi et al., 2021).

### Anti-Biofilm Activity

**FIGURE 7:**
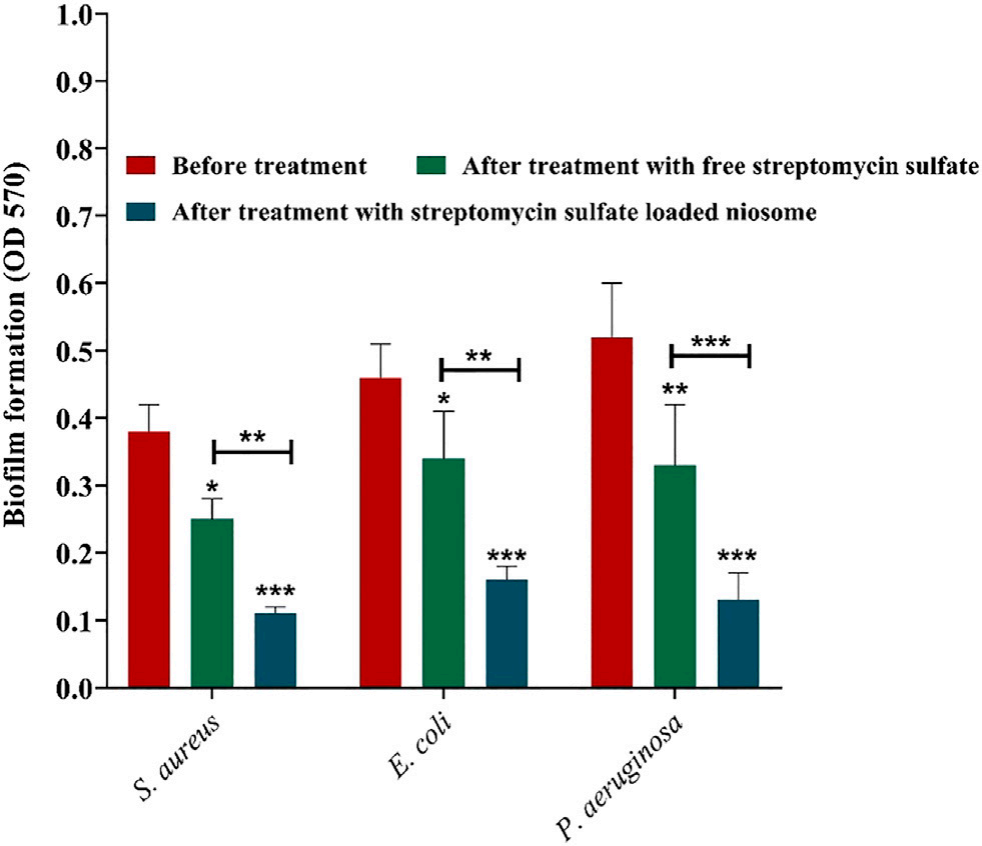
Anti-biofilm activity of free and niosome-encapsulated streptomycin sulfate against selected pathogenic bacterial biofilms at their minimal inhibition concentrations shown in Figure 5. Biofilms were formed in a 96-well microplate and, consequently, treated for 24 h at 37°C. The remaining biofilm was quantified by CV staining and compared with the untreated one. Data represent the mean ± SD (*n* = 3). Error bars represent standard deviations. The levels of significant difference are denoted by *p value < 0.05, **p value < 0.01, and ***p value < 0.001.

Since biofilm is a prevalent factor causing antimicrobial resistance and accounts for 65–80% of all infections (Macià et al., 2018), the fabricated niosomes here were investigated for their efficacy against biofilms of *S. aureus*, *E. coli*, and *P. aeruginosa* with MIC shown in Figure 5. It was revealed that streptomycin-loaded niosomes reduced significantly the preformed biofilm in comparison to the free streptomycin (Figure 7). Previously, it has been reported that niosomal vesicles, due to their cationicity, interact electrostatically with the negatively charged biofilms; the drug can be released into the biofilm structure. Thus, niosomes are excellent carriers for delivery of antimicrobial drugs for eradication of biofilms. Previously, Kashef et al. studied the anti-biofilm effects of ciprofloxacin-containing niosomes against *S. aureus* biofilm and showed that niosome encapsulation reduced the minimum biofilm eradication concentration of ciprofloxacin by 2- to 4-fold compared to free ciprofloxacin (Kashef et al., 2020). In this work, even with 4- to 8-fold lower MIC of the free streptomycin (Figure 5), encapsulation allowed more efficient removal of the biofilm (Figure 7). This result once more demonstrates the power of niosomes.

### Cytotoxicity

**FIGURE 8:**
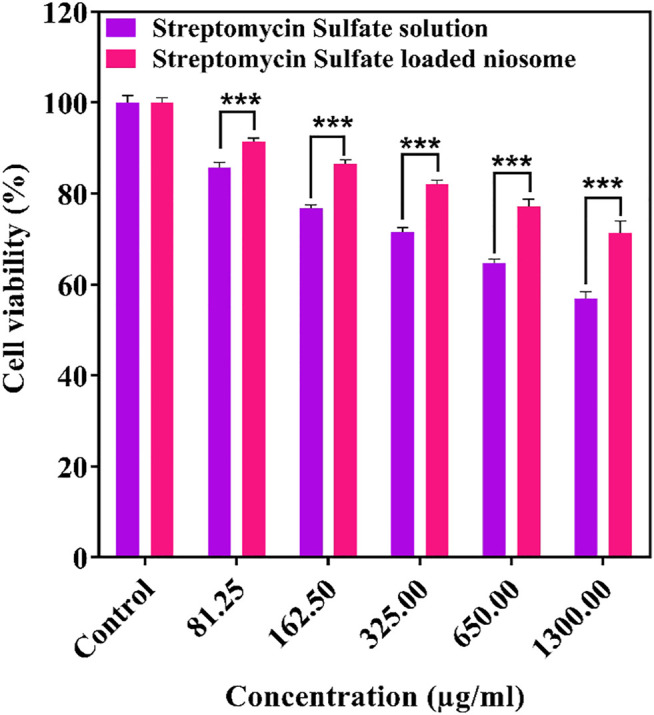
Cytotoxicity of free and niosomal streptomycin sulfate in different concentration against HFF after 24 h. ***p < 0.001, **p < 0.01, *p < 0.05.

The free and encapsulated streptomycin was evaluated for their cytotoxicity toward the HFF using the MTT assay. The cells exposed to medium only were used as control, and their viability was set to 100%. The cytotoxic cutoff was set as 70% of the viable cells in the control. It can be noticed that the niosome-encapsulated streptomycin had low toxicity (cell viability above 70%) to the HFF cells within the tested concentrations up to 1,300 μg/ml after 24 h, whereas free streptomycin exhibited toxicity with a concentration at 1,300 μg/ml (Figure 8). The lower toxicity of the niosome-encapsulated streptomycin than the free drug may be due to the use of surfactants (Span and Tween) in the niosomes, which are highly biocompatible (Marianecci et al., 2010; Shaker et al., 2015). The lower toxicity can also be caused by the lower release of streptomycin from niosome than the free form (Figure 4): after 24-h interaction, only about half of streptomycin was released from niosomes compared with that from the free form. Consequently, slightly higher cytotoxicity was observed for the free form than the niosome form (Figure 8), with about 70% cell viability for the former and 80% for the latter at the highest tested concentration. The low toxicity of niosomal carriers can be an ideal proposition in clinical applications.

## Conclusion

In this study, the optimal niosomal formulation was designed and synthesized for streptomycin sulfate, with the highest encapsulation efficiency but a minimum size and low PDI. The optimized niosomal formulation exhibited controlled drug release and antibacterial effects against both Gram-positive and -negative strains. In addition to increased antibacterial activity of drug-containing nano-carriers, the niosomes showed reduced toxicity to normal cells compared to free streptomycin sulfate. The results of this study can lead to a new therapeutic process in the improvement and treatment of infection. It is envisaged that further *in vivo* studies shall be performed to investigate the function of this nanostructure in treatment of microbial infections.

## Data Availability

The original contributions presented in the study are included in the article/Supplementary Material; further inquiries can be directed to the corresponding authors.
